# Clinical Presentation, Treatment, and Outcomes of 28 Patients With Castleman Disease: A Retrospective Analysis of an Italian Cohort

**DOI:** 10.1002/jha2.70158

**Published:** 2025-11-06

**Authors:** Caterina Cristinelli, Michele Merli, Marco Lucioni, Manuel Gotti, Roberta Sciarra, Sara Rattotti, Federico Carpi, Gianmarco Favrin, Benedetta Bianchi, Giuseppe Neri, Marta Coscia, Marcello Gambacorta, Francesco Passamonti, Marco Paulli, Luca Arcaini

**Affiliations:** ^1^ Department of Molecular Medicine University of Pavia Pavia Italy; ^2^ Hemato‐oncology Department Saint Louis Hospital Paris France; ^3^ Division of Hematology Ospedale di Circolo e Fondazione Macchi Varese Italy; ^4^ Division of Hematology Fondazione IRCCS Ca’ Granda Ospedale Maggiore Policlinico Milano Italy; ^5^ Pathology Unit Fondazione IRCCS Policlinico S. Matteo Pavia Italy; ^6^ Division of Hematology Fondazione IRCCS Policlinico S. Matteo Pavia Italy; ^7^ Department of Medicine and Surgery University of Insubria Varese Italy; ^8^ Pathology Unit Synlab, Castenedolo Brescia Italy; ^9^ University of Milano Milano Italy

## Abstract

**Background:**

Castleman disease (CD) encompasses a range of heterogeneous non‐clonal lymphoproliferative disorders, including unicentric (UCD), and multicentric (MCD) forms. The latter is subdivided into HHV‐8+ MCD, POEMS‐MCD, and idiopathic‐MCD, not otherwise specified (iMCD‐NOS).

**Methods:**

Here we report the clinical characteristics and outcomes of 28 consecutive CD patients, diagnosed in two centers of northern Italy according to recently published diagnostic criteria.

**Results:**

UCD was reported in 12 cases (43%) and MCD in 16 (57%). Among these, 6 (21%) were HHV‐8 positive (1 HIV‐positive and 5 HIV‐negative), and 10 (36%) had iMCD‐NOS. Treatment of UCD consisted of surgical excision in 10/12 cases, resulting in ongoing complete remission in all cases. Single nodal localization favorably affected overall survival (OS) and progression‐free survival (PFS) (*p* < 0.05). Out of 16 MCD patients, 10 had iMCD‐NOS and 6 had HHV‐8+MCD. Anti‐IL‐6 monoclonal antibody was used as first‐line treatment in 5/10 iMCD‐NOS patients, 3 of whom relapsed, although none died. Two out of 6 patients with HHV‐8+ MCD were treated with single‐agent rituximab and one with rituximab plus chemotherapy. UCD patients had significantly better OS and PFS compared to iMCD and HHV‐8+MCD groups (*p* < 0.001).

**Conclusions:**

Our report confirms that UCD, iMCD‐NOS, and HHV‐8+MCD represent distinct clinical entities with different outcomes requiring specific treatment approaches.

**Trial Registration:**

The authors have confirmed clinical trial registration is not needed for this submission.

## 1 Introduction

Castleman Disease (CD) comprises a spectrum of lymphoproliferative disorders with unique histological features at the lymph node biopsy [1]. The clinical presentation varies from asymptomatic isolated nodal lesions to a severe inflammatory disorder that can cause life‐threatening multiple‐organ dysfunction.

Its incidence rate is 21 per million person‐years in the USA. The disease has historically been classified based on histological patterns such as hypervascular (HV) or plasmacytic (PC) [2]. Histological variants are now known to represent an evolving spectrum of the same disease, as many cases exhibit pathological features fluctuating between the two subtypes. The 5th edition of the World Health Organization classification of Haematolymphoid Tumour recognizes three entities: unicentric Castleman disease (UCD), idiopathic multicentric Castleman disease (iMCD), and Kaposi Sarcoma Herpesvirus/Human Herpesvirus 8 (KSHV/HHV‐8)‐associated multicentric Castleman disease (KSHV/HHV‐8+ MCD) [3]. Recently, the International Consensus Classification of Mature Lymphoid Neoplasms [4] inscribed MCD in the HHV‐8–associated lymphoproliferative disorders group.

Diagnosis requires the integration of histological, radiological, clinical‐biological, and virological parameters, and diagnosis and treatment guidelines have been proposed [5, 6, 7].

UCD is a localized disease with a favorable prognosis, mainly occurring in the fourth decade without gender preference [8]. In most cases, the surgical resection leads to complete remission [9]. Most patients present an HV subtype and the clinical presentation generally does not include systemic symptoms, even though an MCD‐like inflammatory syndrome “UCD‐MIS variant” has been described [10]. Even though life expectancy is usually not changed after the diagnosis of UCD, patients are at increased risk of developing autoimmune complications (e.g., paraneoplastic pemphigus [PNP], bronchiolitis obliterans [BO]) [11, 12], vascular neoplasia arising within the lesions of HV follicular hyperplasia (e.g., follicular dendritic cells sarcoma) [13] and AA amyloidosis [14, 15]. Management and course of these complications may diverge from those of UCD, despite its surgical resection, with potentially life‐threatening consequences. Therefore, a systematic screening for such conditions should be conducted at the time of diagnosis.

iMCD represents a polyclonal lymphoproliferative disorder whose etiology remains unknown. Different mechanisms have been proposed to explain it, including auto‐inflammatory, paraneoplastic, and undetermined viral agents. It manifests as a consequence of a rapidly progressing systemic inflammation initially driven by cytokines elevation, including interleukin‐6 (IL‐6) and Vascular Endothelial Growth Factor (VEGF). The self‐sustaining inflammation can lead to severe and potentially fatal dysfunction in multiple organ systems. The classical clinical presentation consists of diffuse edema, peripheral cytopenias, small generalized lymphadenopathies with or without hepato‐splenomegaly, elevation in inflammatory markers, and hypoalbuminemia [5]. According to the clinical presentation, iMCD can be classified into iMCD‐TAFRO (Thrombocytopenia, Ascites, Fever, Reticulin Fibrosis and/or Renal Dysfunction, Organomegaly) [16] or iMCD‐NOS (Not Otherwise Specified). Cases that occur in the context of a POEMS syndrome (Polyneuropathy, Organomegaly, Endocrinopathy, Monoclonal Plasma Cell Disorder, and Skin Changes) are named POEMS‐associated MCD. The 2018 International Consensus for the first‐line treatment of iMCD recommends the use of an anti‐IL6 monoclonal antibody with or without steroids depending on disease severity [7].

The diagnosis of KSHV/HHV8+ MCD relies on the demonstration of in situ HHV8 infection using LNA immunohistochemistry in the context of multiple adenopathies and pathological features consistent with CD [4]. This subtype is more common in HIV‐positive subjects, in which the overall incidence is estimated at 4.3 per 10.000 patient‐year [18]. HHV‐8+ MCD shows a more aggressive behavior than iMCD [19] with recurrent exacerbations characterized by systemic symptoms, CRP elevation, and a high HHV8 viral load in peripheral blood mononucleated cells (PBMCs) [20]. The hemophagocytic syndrome is reported to be the most frequent complication [21]. Patients may suffer from concomitant KSHV‐associated diseases such as KS and non‐Hodgkin's lymphomas, whose presence can negatively impact survival [22, 23]. Despite its remitting‐relapsing behavior, the disease outcome has impressively improved after the introduction of rituximab [24], which proved to be effective also for retreatment [25].

## 2 Materials and Methods

### 2.1 Patients

In the present study, we conducted a retrospective analysis of 28 CD patients, aged > 18 years, whose diagnoses were performed between 2000 and 2022 at the Division of Hematology of Pavia, comprising cases referred from the Division of Hematology of Varese.

All the patients were first searched in the pathology database of Policlinico San Matteo and Ospedale di Circolo‐Fondazione Macchi. The eligible cases were required to have a diagnosis of CD based on the pathological analysis of a lymph node biopsy according to the reported histologic criteria [5, 6]. Formalin‐fixed, paraffin‐embedded (FFPE) biopsies were stained with hematoxylin and eosin (HE) and Giemsa. All cases were reviewed in a multi‐head microscope session by three expert hemopathologists with specific expertise in CD from the Pathology Unit of Policlinico S. Matteo in Pavia. All cases from Ospedale di Circolo‐Fondazione Macchi were centrally reviewed by the same experts. Based on the pathological features, cases were classified into hyaline‐vascular, PC, or mixed subtypes. For all patients with iMCD‐NOS, an infectious, rheumatologic, and histopathologic work‐up was performed to exclude alternative diagnoses and, consequently, cases of “Castleman‐like” lesions. This included HIV, hepatitis B, and C virus, EBV, cytomegalovirus (CMV), *Toxoplasma*, syphilis serology, Quantiferon TB test, antinuclear antibodies (ANA), anti‐extractable nuclear antigens (ENA), and rheumatoid factor, serum protein electrophoresis, and immune‐fixation. For those presenting a monoclonal protein, additional analyses allowed for the exclusion of a clonal disorder (Table S3).

Immunohistochemical analysis of nodal tissue with antibodies against CD20, CD79a, BCL2, CD10, BCL6, CD138, CD3, CD5, κ, λ, IgG, IgG4, Mib1/Ki‐67, CD21, CD23, Cyclin D1, CD68R/PGM1, CD34, HHV8/LNA‐1 (Agilent/Dako, Santa Clara, California, USA) was performed using the automated platform Dako Omnis Envision Flex. IGVH gene rearrangements were investigated through PCR for FR1, FR2, and FR3 using IdentiClone IGH Gene Clonality Assay (Invivoscribe, San Diego, California, USA) on FFPE samples. HHV‐8‐specific staining for LANA‐1 was used to detect HHV‐8‐infected B cells. All cases were tested for Epstein–Barr Virus (EBV) through in situ hybridization (ISH). A total of 21 out of 28 patients have been previously described from a biological and histopathological point of view and reported in a recent analysis from our group [26].

The disease was classified as unicentric in the presence of isolated lymphadenopathy and multicentric when multiple lymph nodes were identified through a radiologic exam performed before the treatment initiation, such as a CT scan and/or 18Ffluoro‐2‐deoxy‐D‐glucose (FDG) positron emission tomography (PET) scan. The final diagnosis was made according to the consensus diagnostic criteria [5, 6], retrospectively applied to the diagnoses made before 2017. For the included patients, we collected a dataset of clinical, biological, and treatment data at the time of the histological diagnosis.

The treatment responses were defined through the integration of biochemical, clinical, and radiological parameters according to the proposed guidelines [6]. For the patients treated with systemic therapy, a radiological evaluation was performed at least 4 weeks after the end of treatment. A progressive disease (PD) was defined when at least one of the parameters was progressive. A partial or complete response (PR, CR) always needed a consensual radiological response.

Patients were followed until July 31, 2023, and we documented the survival status. Survival outcomes were defined as the period from diagnosis to either death or last follow‐up.

The study was conducted within the Comprehensive Register of Lymphoproliferative Disorders (ReLy), approved by the Ethical Committee of Pavia, and according to the Helsinki Declaration of 1975.

### 2.2 Statistical Analysis

Continuous variables are expressed as mean and categorical ones as number of cases (%). Statistical analyses were performed with R statistical software version 3.6.3. The Kaplan–Meier method was used to build survival curves and calculate 2‐ and 5‐year survival rates. The log‐rank test was used to compare survival curves. Univariate Cox regression was used to calculate the hazard ratios (HRs). Variables with *p* < 0.05 by univariate analysis were further analyzed by multivariate Cox regression. A *p* < 0.05 was used as a threshold for statistically significant differences.

## 3 Results

In total, we identified 28 patients, 15 males (54%) and 13 females (46%). All the diagnoses were established by a panel of pathologists and hematologists. The baseline characteristics of the included patients and the initial treatment information are summarized in Table 1. Additional details about biological, clinical and treatment characteristics for each patient are available in the Table S1 of Supporting Information.

**TABLE 1:** Baseline characteristics and first‐line treatment data of 28 patients with Castleman disease.

	All cases	UCD, *n* (%)	iMCD, *n* (%)	HHV‐8+ MCD, *n* (%)
**Number of cases**	28	12/28 (43%)	10/28 (36%)	6/28 (21%)
**Sex (M/F)**	15/13	4/8	5/5	6/0
**Median age (range)**	56 (16–84)	53 (16–69)	58 (40–75)	75 (35–84)
**Histological classification**				
HV	12/28 (43%)	10/12 (83%)	1/10 (10%)	1/6 (17%)
PC	5/28 (18%)	0/12	4/10 (40%)	1/6 (17%)
Mixed	11/28 (39%)	2/12 (17%)	5/10 (50%)	4/6 (66%)
**HIV‐positive**	1/28 (4%)	—	—	1/6 (20%)
**HHV8‐positive** **‐Immunohistochemistry** **‐Blood PCR**	6/28 (21%)	—	—	6/6 (100%) 5/6 (83%) 5/5 (100%)
**ECOG PS ≥ 2**	10/28 (36%)	1/12 (8%)	4/10 (40%)	5/6 (83%)
B symptoms*	8/27 (30%)	1/12 (8%)	3/9 (33%)	4/6 (67%)
**Hb < UNL**	13/27 (48%)	2/11 (18%)	5/10 (50%)	5/5 (100%)
**PLT < UNL**	6/27 (22%)	1/11 (9%)	1/10 (10%)	4/6 (67%)
**Hypoalbuminemia**	12/27 (44%)	2/11 (18%)	4/10 (40%)	6/6 (100%)
**CRP > UNL**	11/24 (46%)	0/9 (0%)	5/10 (50%)	6/6 (100%)
**IL‐6 > UNL**	13/16 (80%)	4/6 (67%)	6/7 (86%)	3/3 (100%)
**IgG > UNL**	10/20 (50%)	0/7 (0%)	6/9 (67%)	4/4 (100%)
**Monoclonal serum protein**	10/26 (39%)	2/12(17%)	5/8 (60%)	3/6 (50%)
**Complications**				
Hepato/splenomegaly	11/28 (39%)	2/12 (17%)	4/10 (40%)	5/6 (83%)
Edema/effusion	6/28	2/12 (17%)	2/10 (20%)	2/6 (33%)
Lung	0/28	0/12	0/10	0/6
Skin	0/28	0/12	0/10	0/6
Kidney (eGFR < 60 mL/min)	4/18	0/7	0/4	4/6 (67%)
Proteinuria	8/25 (32%)	3/12 (25%)	5/7 (70%)	4/6 (67%)
PNP	0/28	0/12	0/10	0/6
Peripheral neuropathy	2/28 (7%)	0/12	2/10 (20%)	0/6
DAT+	3/28 (11%)	0/12	0/10	3/6 (50%)
HLH	2/28 (7%)	0/12	0/10	2/6 (33%)
**Autoimmune disease°**	7/28 (25%)	1/12 (8%)	3/10 (30%)	3/6 (50%)
**Associated solid neoplastic condition**	8/28 (29%)	2/12 (17%)	3/10 (30%)	3/6 (50%)
**Lymphoproliferative disease**	3/28 (11%)	0/12	3/10 (30%)	0/6
**Secondary amyloidosis**	2/28 (7%)	2/10 (20%)	0/10	0/6
**POEMS**	0/28	0/12	0/10	0/6
**TAFRO Syndrome**	0/28	0/12	0/10	0/6
**Bone marrow alterations**				
**CD specific infiltration**	2/14 (14%)	0/4	0/7	2/3 (67%)
**Polyclonal plasmacytosis**	7/14 (50%)	1/4 (25%)	4/7 (57%)	2/3 (67%)
**1st line treatment**				
**Surgical resection**	10/28 (36%)	10/12 (83%)	—	—
**Watch and wait**	1/28 (3.5%)	1/12 (8%)	—	—
**Anti‐IL6 Ab**	5/28 (18%)	—	5/10 (50%)	—
**Rituximab single agent**	2/28 (7%)	—	—	2/6 (33%)
**Rituximab + chemo**	1/28 (3.5%)	—	—	1/6 (17%)
**Chemotherapy**	2/28 (7%)	—	1/10 (10%)	1/6 (17%)
**Steroids**	3/28 (11%)	1/12 (8%)	2/10 (20%)	—
**Not treated**	4/28 (14%)	—	2/10 (20%)	2/6 (33%)

Abbreviations: CD, Castleman disease; CMV, cytomegalovirus; DAT: direct antiglobulin test; EBV, Epstein–Barr virus; GFR, glomerular filtration rate; Hb, hemoglobin; HHV8, human herpes virus‐8; HIV, human immunodeficiency virus; HLH, hemophagocytic lymphohistiocytosis; HV, hypervascular; IgG, Immunoglobulin G; IL‐6, interleukin‐6; PC, plasmacytic; PNP, paraneoplastic pemphigus; UNL, upper normal limit; WBC, White Blood Count.

**FIGURE 1:**
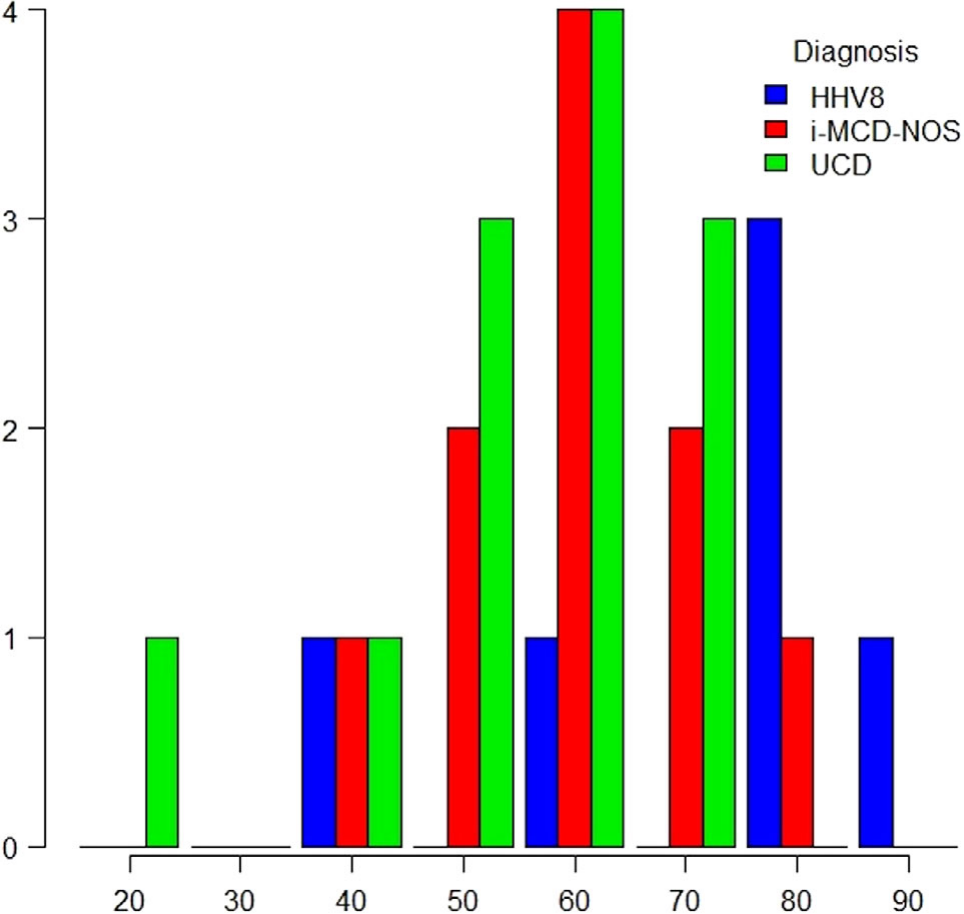
Distribution of diagnosis of Castleman disease per age class.

Out of the total 28 cases, the disease was unicentric in 12 cases (43%) and multicentric in 16 (57%). Among these, 6 (21%) were HHV‐8 positives, and 10 (36%) had iMCD. The median age at diagnosis was 56 (range 16–84). The diagnoses distribution according to different age classes is depicted in Figure 1.

UCD showed a female predominance and the mean age at diagnosis was 53 (range 16–69). All the patients had a unicentric presentation, and no oligocentric forms were found. Lesions localization was: latero–cervical (*n* = 2), submandibular (*n* = 2), mediastinal (*n* = 1), retroperitoneal (*n* = 2), inguinal (*n* = 2), mesenteric (*n* = 2), pelvic (*n* = 1).

The enlargement of isolated adenopathy was the most frequent initial symptom (10/12, 84%). Patients generally had an excellent performance status. IL‐6 was above the upper normal limit (UNL) in 4/6 patients (67%), and the median value was 6.4 pg/mL (range 0.1–53). The median hemoglobin and platelet levels were in normal ranges. A bone marrow biopsy was performed in five cases, and one case showed a plasma cellular polyclonal infiltrate. No patient had a UCD‐related complication at the time of diagnosis. The most frequent histologic variant was the HV (10/12, 83%). Two patients with AA amyloidosis presented a mixed histological variant (2/12, 17%). Other concomitant clinical conditions were hepatic carcinoma, ovarian cystadenoma, sickle cell anemia, and a previous idiopathic paralysis of the VII and III pairs of cranial nerves.

The treatments consisted of surgical excision (10/12), steroids (1/12), and watch and wait (1/12). In all the cases the disease was potentially entirely resectable. At the time of the data analysis, all the treated patients were in CR. The non‐treated patient was alive with stable disease. One patient died for causes other than CD.

In univariate analysis, a single nodal localization was significantly associated with better progression‐free survival (PFS) (HR = 0.083; 95% CI: 0.0106–0.646; *p* = 0.018) and with a borderline longer overall survival (OS) (HR = 0.165; 95% CI: 0.019–1.380; *p* = 0.096).

Considering MCD patients (*n* = 16), 10 were diagnosed as iMCD‐NOS and 6 as HHV8+ MCD. In the iMCD‐NOS subgroup, the median age at diagnosis was 58 (range 40–75) without a gender predominance. Four cases exhibited a PC histology, five mixed, and one HV. Histological grading for iMCD‐NOS patients is detailed in Table S2.

Almost half of the patients presented an altered general status reflected by an ECOG performance status ≥ 2. The most frequent clinical presentations included B symptoms (4/10, 40%) and hepato‐splenomegaly (4/10, 40%). Laboratory tests revealed proteinuria (5/7), hypergammaglobulinemia (6/9) with a serum monoclonal protein in more than half of the patients (5/8), and anemia (5/10). Four cases showed a polyclonal plasma cellular infiltrate at the bone marrow biopsy. ESR and IL‐6 levels were above the UNL in all the evaluated patients. The median level of IL‐6 was 20.6 pg/mL (range 1.59–83). The patient with HV histology presented a less symptomatic disease, with a slight elevation of the inflammatory markers, and no dysproteinemia or fluid retention. Three patients had a rheumatological disorder or alteration not consistent with a CD exclusion criterion (one isolated positivity of anti‐ANA and anti‐SCL70 antibodies, one Hashimoto's thyroiditis and Reynaud syndrome, one limited cutaneous systemic sclerosis). Three patients developed a clonal hematological malignancy (one bone plasmacytoma, one extranodal marginal zone lymphoma, one symptomatic IgG‐kappa multiple myeloma) 1, 2, and 8 years after the diagnosis of iMCD‐NOS. The bone plasmacytoma was one of the manifestations of a multiple tumor syndrome comprising glioblastoma multiforme and renal oncocytoma. One patient had a concomitant undifferentiated rectal carcinoma. No cases of TAFRO syndrome were described. The two patients who developed a plasma cell neoplasm did not fulfil the necessary criteria for a POEMS‐associated CD.

Anti‐IL‐6 monoclonal antibody was used as first‐line treatment in five patients (5/10: Siltuximab *n* = 4; Tocilizumab followed by Siltuximab *n* = 1). In one case steroidal therapy was added due to disease severity. In three patients, the disease progressed after 6, 24, and 38 bi‐weekly administrations, respectively. As a result, these patients required subsequent cyclophosphamide or anthracyclines‐based chemotherapy with or without rituximab. One patient developed a multiple myeloma while in CR after 118 injections and one patient maintained an on‐therapy PR after 33 cycles. In one case, the patient achieved a complete transitory response even though the pre‐treatment IL‐6 serum levels were not increased. All patients treated with anti‐IL‐6 antibodies were alive at the data analysis (*p* = 0.18). Other used first‐line therapies were: corticosteroids (2/10), cyclophosphamide‐based chemotherapy (1/10), and Chlorambucil plus corticosteroids (1/10). One patient did not receive treatment.

Six patients were diagnosed with HHV8+ MCD. One of them was HIV+, five HIV−. Circulating HHV‐8 DNA was detectable in all the tested patients (5/5). The median age at diagnosis was 75 (range 35–84) and all the patients were males. The histological analysis revealed a PC or mixed pattern in five cases and HV in one. At the diagnosis, all the patients were markedly symptomatic. Major clinical features included B symptoms (4/6), hepato‐splenomegaly (5/6), and fluid retention (2/6). The clinical presentation was complicated by hemophagocytic lymphohistiocytosis (HLH) in two cases. Two patients had a concomitant KS (one HIV‐positive and one HIV‐negative). Biological alterations included anemia (6/6), thrombocytopenia (4/6), renal failure (4/6), proteinuria (4/6), and hypergammaglobulinemia (4/4). A direct antigenic test (DAT) was positive in three cases, with an associated hemolytic anemia in 2. IL‐6 was augmented in both the tested patients with a median level of 55.2 pg/L. A bone marrow CD‐specific infiltration was documented in two cases (2/3, 67%): in one case a polyclonal plasmacytosis and HHV8+ plasmablasts coexisted with images of hemophagocytosis; in another, an EBV immunostaining positivity revealed a concomitant EBV coinfection. The two patients whose disease was complicated by HLH presented a highly aggressive clinical course with early onset terminal multi‐organ failure and concomitant viral reactivations. Out of six patients, two had colon adenomas, one had an autoimmune diathesis with alopecia areata, allergic asthma, and eczematous dermatitis, one had vitiligo, and one had a history of lung cancer.

**FIGURE 2:**
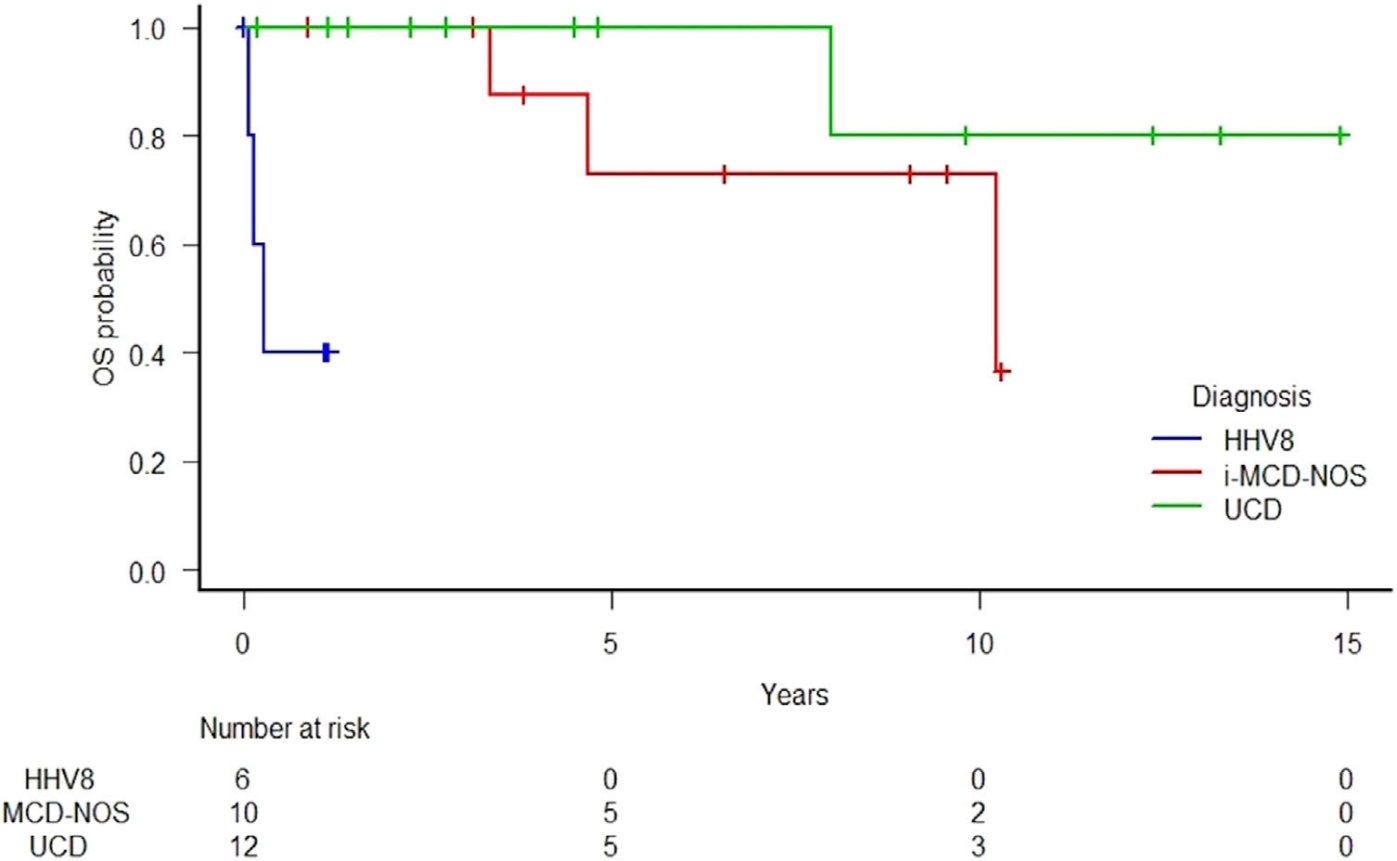
Overall survival according to disease category (iMCD‐NOS vs UCD vs HHV8+ MCD).

**FIGURE 3:**
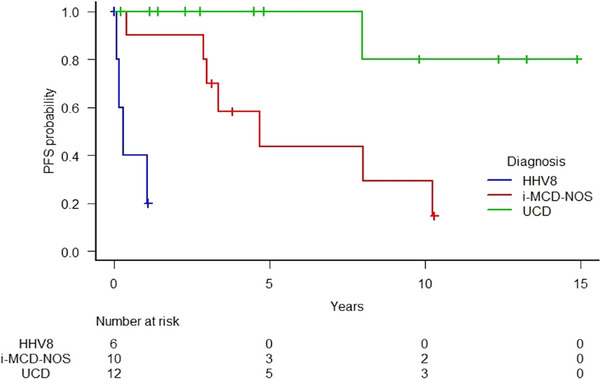
Progression‐free (B) curves of Castleman disease patients stratified by categorical diagnosis (iMCD‐NOS vs UCD vs HHV8+ MCD).

In first line, two patients were treated with rituximab single agent: one obtained a CR after 4 weekly doses but relapsed 8 months later, necessitating admission to intensive care for hepatic and renal failure. A retreatment with the same weekly regimen was attempted and the results were ongoing at the moment of data analysis; the other died after a single dose as a result of HLH. The HIV+/KS+ patient received a combination of rituximab and liposomal doxorubicin obtaining a CR after 4 and 12 doses respectively. One patient received treatment for KS, which included cryotherapy, liposomal doxorubicin, paclitaxel, etoposide, and local radiotherapy. The patient showed a response on the CD but was then lost to follow‐up. Two patients were not considered susceptible to any treatment due to HLH complication (1/6) and infectious comorbidities (1/6) and they died with active disease. UCD patients had significantly better OS (*p* < 0.001) and PFS (*p* < 0.001) if compared to iMCD‐NOS and HHV8+ MCD groups (Figure 2 and 3).

With a median follow‐up of 3.6 years, the 5‐year OS and PFS of the whole cohort were 75.8% (95% CI: 49.7–89.7), and 73.9% (95% CI 48.3–87.4), respectively. HHV8+MCD group showed the worst OS and PFS (40% at 1 year) (Table 2). Based on univariate logistic regression, besides CD groups, the prognostic factors influencing the PFS were GFR < 60 mL/min (*p* = 0.002), hepatosplenomegaly (*p* = 0.013), ECOG ≥ 2 (*p* = 0.026), hypoalbuminemia (*p* = 0.035), thrombocytopenia (*p *= 0.048), B symptoms (*p* = 0.041). At the multivariate Cox regression, only GFR < 60 mL/min (HR 22.39, 95% CI: 2.16–231.4, *p* = 0.009) and ECOG ≥ 2 (HR 8.23, 95% CI: 1.04–65.11) were demonstrated to significantly affect PFS.

**TABLE 2:** Patient outcomes according to different Castleman disease entities.

	Median follow‐up	2‐Year PFS (%)	2‐Year OS (%)	5‐Year PFS (%)	5‐year OS (%)
**All patients**	3.6 y	84.7	88.7	73.1	75.9
**UCD**	3.9 y	100	100	100	100
**iMCD‐NOS**	5.6 y	90	100	43.8	72.9
**HHV8+MCD**	78 d	Estimated 1‐year PFS (%)		Estimated 1‐year OS (%)	
		40		40	

Abbreviations: d: days, OS: overall survival, PFS: progression‐free survival, y: years.

## 4 Discussion

CD encompasses a range of non‐clonal lymphoproliferative disorders characterized by typical nodal histopathology and different clinical presentations and outcomes.

In the present study, we analyzed 28 consecutive patients with CD, diagnosed over 20 years in two Hematology Units in Italy, and we found different features and outcomes.

Patients with a diagnosis of UCD revealed a prevalence of an HV histology, consistent with other reports from the USA [27], China [28], and France [19]. The clinical presentation was mostly driven by the appearance of an isolated enlarged adenopathy, without associated inflammatory syndrome, although IL‐6 plasma levels were frequently elevated. The median age at diagnosis was higher than in the UCD French cohort [29]. Unlike other populations, we did not observe any usual complications at diagnosis, such as PNP [30]. Together with the absence of an inflammatory profile and oligocentric presentation, this is probably responsible for the excellent outcome of our cohort, even better than previously reported [19, 27, 31]. The clinical association between AA amyloidosis and CD has already been described, to the point that patients with UCD or MCD complicated by organ failure should prompt consideration of concurrent AA amyloidosis [14, 15]. In our cohort, two patients had a previous diagnosis of AA amyloidosis. One of them presented a renal and hepatic involvement with consequent renal failure, nephrotic range proteinuria, hepato‐splenomegaly, and peripheral edema. After the CD surgical excision, the renal function underwent a transient recovery and the plasma level of the serum amyloid rapidly decreased. Six months after the nodal excision, a rebound of the renal disease occurred requiring hemodialysis and kidney transplantation. Although it has been reported that surgical resection can reverse the associated symptoms [32], the reappearance of renal insufficiency suggests the the establishment of a self‐sustaing mechanism arising after CD. Given the absence of oligocentric presentations, complete surgical excision was the treatment of choice for almost all the patients, achieving a CR in all cases.

In the iMCD‐NOS cohort, we confirmed the prevalence of PC or mixed histology. Similarly to larger series and recently published meta‐analysis [19, 33, 34] the median age at diagnosis was older than for the UCD variant. Compared to UCD, most patients were symptomatic at diagnosis. Polyclonal plasmacytosis was the most common feature at the bone marrow biopsy, often accompanied by an increase in IgG and IgA circulating levels, as already reported [35].

The clinical overlapping between POEMS syndrome and MCD makes the differential diagnosis sometimes difficult. Though no cases of POEMS‐related CD were reported in our series, one patient exhibited disseminated adenopathies associated with plasma cellular dyscrasia, peripheral edemas, elevation of VEGF circulating levels, Hashimoto's thyroiditis, Reynaud syndrome with acrocyanosis and hirsutism. In the absence of peripheral neuropathy, a diagnosis of POEMS could not be retained, and the case was finally classified as iMCD‐NOS. Of note, the patient was refractory to the anti‐IL‐6 treatment and responded partially to a rituximab–cyclophosphamide‐based regimen. In another case, MCD‐POEMS differential diagnosis was challenging. The patient presented a monoclonal protein, sensitive‐motor neuropathy, and a CD nodal histology. However, in the absence of minor criteria the MCD‐POEMS diagnosis could not be retained.

CD is associated with malignancies and several hypotheses have been proposed to explain this. These include the production of cytokines by malignant cells leading to CD, a common genetic mutation, promotion of malignant transformation by cytokine release, and susceptibility to malignant transformation due to cytotoxic therapy. Though there is uncertainty about the increased risk of solid cancer, UCD, HHV8+ MCD, and iMCD seem to be associated with an increased risk of hematological malignancies [36]. In our cohort, we described an association between CD and hematological and non‐hematological malignancies. All the hematological malignancies arose after the diagnosis of CD but the small number of patients does not allow for consideration about the relationship between CD and NHL or plasma cell neoplasms.

Autoimmune diseases are known to be associated with CD. Different hypotheses have been proposed to explain this connection, including the release of IL‐6, expansion of B‐cells producing autoantibodies, and disease exacerbation by antigen‐presenting cells [26, 37, 38, 39, 40, 41]. Out of the total series, seven patients showed associated autoimmune conditions (1 UCD, 3 iMCD‐NOS, 3 HHV8+ MCD) in the form of clinical manifestations and/or of circulating auto‐antibodies.

Anti‐IL 6 treatment is considered the first‐line option for the treatment of iMCD‐NOS. A Phase II study conducted on 140 iMCD patients reported durable tumor and symptomatic responses in 34% of the patients in the siltuximab group, without additional toxicity [42]. Out of 10 iMCD‐NOS patients, 5 received an IL‐6‐directed treatment and all were alive at the data cut‐off. Due to the limited number of patients who were treated with anti‐IL6, along with the excellent outcome, it was not possible to determine if certain risk factors could significantly affect the treatment response. However, we observed that low levels of IL‐6 did not hinder the achievement of a clinical response. The 2‐year OS of the iMCD‐NOS cohort confirmed the excellent outcome already reported in the French population [19] and the 5‐year OS was consistent with what was reported by a Korean series presenting more similar numbers and follow‐up duration [43]. The difference between the 5‐year OS and PFS reflects the relatively favorable prognosis of this clinicopathological subtype, some patients being alive with active disease or a PR at the data analysis.

HHV8+ MCD patients were significantly older than those in the UCD and iMCD‐NOS groups. Moreover, the median age of the HHV8+ cohort (75 years) was considerably higher compared to that reported in the largest US series (44 years) [22] and in the recent meta‐analysis (48 years) [33]. In accord with other described populations [21, 34], all the patients were male and the histology was prevalently PC or mixed. Clinically, the disease tended to be more aggressive, with more frequent systemic symptoms, peripheral cytopenias, hepato‐splenomegaly, renal impairment, and hypoalbuminemia. Two cases of non‐CD HHV8‐associated disorders, notably KS, were detected, irrespective of the HIV status. Rituximab in the first line setting leads to a 70% increase in the 5‐year OS [44]. Two of the three patients treated with Rituximab‐based protocols obtained a CR. One patient, whose clinical presentation was dominated by a rapidly evolving HLH, was rituximab‐refractory. Surprisingly, the HIV‐positive patient had a non‐aggressive clinical presentation and an excellent outcome. Differently from other series [19, 22], where generally favorable long‐term outcomes were reported, the prognosis of our subpopulation was extremely poor. This is probably related to the older age and to the relatively frequent association with HLH.

By univariate and multivariate Cox regression analyses, a renal function impairment (GFR < 60 mL/min) and a poor performance status (ECOG ≥ 2) independently affected PFS in the whole cohort, similar to what was reported by a large retrospective Chinese analysis [31].

In conclusion, this is one of the largest Italian series reported to date based on clinical and pathological data of patients affected by CD. Our report confirms that UCD, iMCD‐NOS, and HHV‐8+MCD represent three distinct clinical entities with different outcomes that need specific treatment approaches. In our series, UCD showed a favorable clinical behavior and prognosis when compared to iMCD‐NOS, and HHV8+MCD was associated with the poorest outcome. Adequate clinical recognition and classification, together with an expert pathological review, are fundamental for the correct management of this challenging disease. A collective effort to find an appropriate etiologic treatment is necessary to ameliorate prognosis, especially in immunodeficiency‐related forms.

## Author Contributions

**Caterina Cristinelli**: design of the research study, data collection, data analysis, writing – original draft, writing – review and editing. **Michele Merli**: design of the research study, data collection, data analysis, writing – review and editing. **Marco Lucioni**: histologic diagnosis, data analysis, writing – review and editing. **Manuel Gotti**: data collection, writing – review and editing. **Roberta Sciarra**: data collection, writing – review and editing. **Sara Rattotti**: data collection, writing – review and editing. **Federico Carpi**: data collection, writing – review and editing. **Gianmarco Favrin**: data collection, writing – review and editing. **Benedetta Bianchi**: data collection, writing – review and editing. **Giuseppe Neri**: design of the research study, data collection, writing – review and editing. **Marta Coscia**: writing – review and editing, supervision. **Francesco Passamonti**: writing – review and editing, supervision. **Marcello Gambacorta**: writing – review and editing, supervision. **Marco Paulli**: histologic diagnosis, writing – review and editing, supervision. **Luca Arcaini**: design of the research study, data analysis, writing – review and editing, supervision. All authors have read and agreed to the submitted version of the manuscript.

## Conflicts of Interest

**Michele Merli**: Regeneron Inc. (Consultancy). **Marco Paulli**: Recordati Rare diseases (Honoraria). **Luca Arcaini**: EUSA Pharma, Novartis (Honoraria); Roche, Janssen‐Cilag, Verastem, Incyte, EUSA Pharma, Celgene/Bristol Myers Squibb, Kite/Gilead, ADC Therapeutics, Novartis (Participation on a Data Safety Monitoring Board or Advisory Board); Roche (Support for attending meetings and/or travel).

## Supporting information




**Table S1**. Most relevant clinical, biological, and treatment data for all the included patients.

**Table S2**. Histopathological grading for all iMCD‐NOS patient according to Fajgenbaum et al. *Blood* 129, 1646–1657 (2017).

**Table S3**. Summary of bone marrow characteristics, kappa/lambda nodal immunostaining, and BCR rearrangement analysis for patients with iMCD‐NOS and M protein, used for ruling out diagnoses other than Castleman disease or Castleman‐like lesions.

## Data Availability

All data shown in the present study are available on request from the corresponding author.
