# Supplementary material for: Clinical Presentation, Treatment, and Outcomes of 28 Patients With Castleman Disease: A Retrospective Analysis of an Italian Cohort
Source: EJHaem. 2025 Nov 6;6(6):e70158. doi: 10.1002/jha2.70158 (PMC12591177; doi:10.1002/jha2.70158)
Supplement: Supplementary file 1 — Table S1. Most relevant clinical, biological, and treatment data for all the included patients. [file JHA2-6-e70158-s002.docx]

| Case N. | Sex | Age | Diagnosis | Histology | Bone marrow biopsy | ECOG PS | CRP (mg/dl) | Hb (g/dL) | WBC (G/L) | PLT (G/L) | Albumine (g/dL) | eGFR (ml/min) | M  protein | IgG (mg/dL) | VEGF | IL-6 (pg/ml) | B symptoms | Hepato-splenomegaly | Fluid retention | Skin reactions | HIV (serology) | Autoimmune disorders | UCD complications | Lymphoproliferative Disorders | First-line treatment | Treatment response | Last follow-up | Disease status at last follow-up | Outcome | Cause of death |
| --- | --- | --- | --- | --- | --- | --- | --- | --- | --- | --- | --- | --- | --- | --- | --- | --- | --- | --- | --- | --- | --- | --- | --- | --- | --- | --- | --- | --- | --- | --- |
| **1** | F | 38 | UCD | HV | NA | 0 | 0,7 | 13,6 | 7,1 | 281 | 4,1 | NA | No | NA | NA | 0,1 | No | No | No | No | Negative | No | No | No | Surgery | CR | 10/10/18 | CR | Alive | // |
| **2** | M | 84 | HHV-8+MCD | Mixed | NA | 2 | 3,45 | 11,2 | 5,6 | 164 | 3,1 | 23,5 | Yes | 1780 | NA | 101 | No | No | No | No | Negative | No | // | No | // | // | 23/05/12 | Active Disease | Dead | Infectious complication* |
| **3** | F | 75 | iMCD-NOS | HV | NA | 0 | 0,26 | 9,3 | 4,8 | 182 | 4,2 | NA | NA | 1630 | NA | 10,74 | No | No | No | No | Negative | No | // | Extranodal Marginal Zone Lymphoma° | Chlorambucil 4 mg/d + Prednisone 12.5 mg/d | SD | 17/11/11 | Active Disease | Dead | Not known |
| **4** | F | 42 | UCD | HV | Negative for CD localization; no fibrosis | 0 | NA | 11,4 | 4,3 | 154 | 4,1 | NA | No | NA | NA | 6,7 | No | No | No | No | Negative | No | No | No | Prednisone 37.5 mg/d | CR | 15/05/13 | CR | Alive | // |
| **5** | M | 75 | HHV-8+MCD | Mixed | CD localization: plasma cells (30%), and B-cells HHV8+ (<10%); no fibrosis | 4 | 25,7 | 7,1 | 5,5 | 16 | 1,3 | 43 | Yes | 1770 | 173 | 67,76 | Yes | Yes | Yes | No | Negative | No | // | No | Rituximab 375 mg/mq | SD | 12/12/20 | Active Disease | Dead | Multiorgan failure in the context of hemophagocytosis |
| **6** | M | 50 | UCD | HV | NA | 0 | 0,08 | 15,2 | 5,9 | 295 | 4,1 | NA | No | NA | 1295 | 1,09 | No | No | No | No | Negative | No | No | No | Surgery | CR | 14/03/23 | CR | Alive | // |
| **7** | M | 52 | UCD | HV | NA | 0 | 0,2 | 14,3 | 4,5 | 152 | 4,0 | 109,2 | No | 1379 | NA | NA | No | No | No | No | Negative | No | No | No | Surgery | CR | 02/07/23 | CR | Alive | // |
| **8** | F | 54 | UCD | Mixed | NA | 1 | NA | NA | NA | NA | NA | NA | IgG-kappa | NA | NA | NA | No | Yes | Yes | No | NA | No | AA Amyloidosis | No | Surgery | CR | 12/07/11 | CR | Dead | Not known |
| **9** | M | 55 | iMCD-NOS | PC | NA | 0 | 16,4 | 12,2 | 7,6 | 425 | 3,2 | 91,68 | No | 1622 | NA | 83 | No | No | No | No | Negative | No | // | No | Siltuximab | PR | 31/07/23 | PR | Alive | // |
| **10** | F | 16 | UCD | HV | NA | 0 | 0,2 | 13,9 | 7,4 | 277 | 4,1 | 125,8 | No | 1430 | NA | NA | No | No | No | No | Negative | No | No | No | Surgery | CR | 31/07/23 | CR | Alive | // |
| **11** | M | 74 | HHV-8+MCD | PC | CD localization (20%), HHV8+ cells; no fibrosis | 1 | 1,6 | 9,5 | 3,6 | 50 | 3,7 | 51,6 | No | 1998 | NA | NA | No | Yes | No | No | Negative | No | No | No | Rituximab 375 mg/mq | CR | 31/07/23 | Active Disease | Alive | // |
| **12** | M | 43 | UCD | HV | NA | 0 | 0,02 | 14,9 | 8,2 | 210 | 4,7 | 105 | No | 1030 | NA | 6,02 | No | No | No | No | Negative | No | No | No | Surgery | CR | 05/05/21 | CR | Alive | // |
| **13** | F | 53 | UCD | HV | Negative for CD localization; polyclonal plasma cells (10%) and reactive B and T lymphocytes (10%); no fibrosis | 0 | NA | 13,6 | 2,5 | 196 | 3,9 | 89 | No | 1220 | 175 | 13,59 | No | No | No | No | Negative | No | No | No | Watch and wait | SD | 15/12/21 | Active Disease | Alive | // |
| **14** | F | 56 | UCD | HV | Negative for CD localization; no fibrosis | 0 | 0,34 | 13,7 | 3,7 | 197 | 4.2 | 97,7 | No | 1340 | NA | NA | No | No | No | No | Negative | No | No | No | Surgery | CR | 06/06/22 | CR | Alive | // |
| **15** | F | 61 | UCD | Mixed | NA | 3 | 0,03 | 11,2 | 6,7 | 642 | 1,4 | NA | Yes | NA | 1062 | 53 | NA | Yes | Yes | No | Negative | No | AA Amyloidosis | No | Surgery | CR | 04/10/22 | CR | Alive | // |
| **16** | M | 60 | iMCD-NOS | PC | Negative for CD localization; no fibrosis | 2 | 1,2 | 14,2 | 5,4 | 201 | 4,3 | 74,8 | No | 1918 | NA | NA | No | No | No | No | Negative | ANA+1:640, anti-SCL70+ | // | No | Prednisone 12.5 mg/d | SD | 05/10/20 | Active Disease | Alive | // |
| **17** | M | 50 | iMCD-NOS | PC | NA | 2 | NA | 17,4 | 6,6 | 275 | 4,7 | NA | IgG-lambda | 1430 | NA | NA | NA | No | No | No | Negative | No | // | Bone plasmacytoma^§^ | // | // | 07/01/06 | Active Disease | Dead | Not known |
| **18** | M | 60 | iMCD-NOS | Mixed | Negative for CD localization; reactive B and T lymphocytes (10%) and polyclonal plasma cells (<5%); no fibrosis | 2 | 0,71 | 13,9 | 7,5 | 368 | 4,5 | 99 | IgG-lambda +  IgG kappa | 948 | 530 | 1,59 | Yes | Yes | No | No | Negative | No | // | No | Siltuximab | PD | 28/10/21 | CR | Alive | // |
| **19** | M | 35 | HHV-8+MCD | Mixed | NA | 2 | 7 | 11,3 | 2,2 | 129 | 3,0 | NA | No | 2230 | NA | NA | Yes | Yes | No | KS | Negative | AHIA and ITP (ES); alopecia areata; eczematous dermatitis; bronchial asthma | // | No | Cryotherapy + RT  Liposomal Doxorubicin, Taxol, Etoposides | SD | 08/04/11 | Active Disease | Lost to follow-up | // |
| **20** | M | 65 | iMCD-NOS | Mixed | Negative for CD localization; reactive B and T lymphocytes (<10%); no fibrosis | 0 | 1,5 | 10,3 | 7,8 | 450 | 3,9 | 77 | IgG-lambda | 3020 | NA | 20,6 | No | No | No | No | Negative | No | // | IgG-k Multiple myeloma^#^ | Siltuximab | CR | 23/11/22 | CR | Alive |  |
| **21** | M | 79 | HHV-8+MCD | Mixed | NA | 2 | 8,93 | 8 | 2,8 | 135 | 3,5 | 52 | No | NA | NA | NA | Yes | Yes | Yes | No | Negative | Vitiligo | // | No | // | // | 01/12/18 | Active Disease | Dead | Multiorgan failure in the context of hemophagocytosis |
| **22** | F | 69 | UCD | HV | NA | 0 | 0,31 | 13,7 | 6,5 | 344 | 4,2 | 80,2 | No | 1560 | NA | NA | Yes | No | No | No | Negative | ANA+ 1:160 | No | No | Surgery | CR | 08/09/22 | CR | Alive | // |
| **23** | M | 67 | UCD | HV | Negative for CD localization; no fibrosis | 0 | 0,33 | 16,3 | 5,5 | 141 | 4,1 | 108,1 | No | 1310 | NA | NA | No | No | No | No | Negative | N | No | No | Surgery | CR | 20/10/22 | CR | Alive | // |
| **24** | M | 57 | HHV-8+MCD | Mixed | Negative for CD localization at diagnosis; reactive B and T lymphocytes (<10%) and polyclonal plasma cells (<10%); loose perivascular reticulin fibrosis | 2 | 15,3 | 11,6 | 10,3 | 228 | 3,1 | 98 | Yes | NAment d | 2718 | 42,7 | Yes | Yes | No | No | Positive | AHIA | // | No | Liposomal Doxorubicin 20 mg/mq + Rituximab 375 mg/mq | CR | 13/10/22 | CR | Alive | // |
| **25** | F | 47 | iMCD-NOS | Mixed | Negative for CD localization; reactive T lymphocytes (5%) and polyclonal plasma cells (<10%); no fibrosis | 3 | 10,7 | 8,4 | 7,9 | 134 | 2,4 | NA | No | NA | NA | 76 | Yes | Yes | Yes | No | Negative | No | // | No | Tocilizumab + Methylprednisolone followed by Siltuximab | CR | 10/10/22 | CR | Alive | // |
| **26** | F | 40 | iMCD-NOS | PC | Negative for CD localization; no fibrosis | 0 | 0,1 | 11 | 5,1 | 214 | 4,0 | 70,34 | IgA-lambda | 1290 | NA | NA | No | No | No | No | Negative | No | // | No | CVP | PR | 01/07/23 | PR | Alive | // |
| **27** | F | 56 | iMCD-NOS | Mixed | Negative for CD localization; reactive T lymphocytes (5%) and polyclonal plasma cells (<10%); no fibrosis | 0 | 4,62 | 13,6 | 10,9 | 230 | 3,4 | 61 | IgG-kappa | 2107 | 2210 | 24,7 | Yes | Yes | Yes | Acrocyanosis,  Hirsutism | Negative | Hashimoto's thyroiditis,  Raynaud's phenomenon | // | No | Siltuximab | PD | 06/06/23 | PR | Alive | // |
| **28** | F | 65 | iMCD-NOS | Mixed | Negative for CD localization; polyclonal plasma cells (<10%); no fibrosis | 0 | 0,10 | 14,9 | 8,4 | 355 | 4,2 | NA | NA | 2350 | NA | 3,97 | No | Yes | No | No | Negative | Limited cutaneous systemic sclerosis | // | No | Low-dose steroids | SD | 08/02/17 | Active Disease | Dead | Not known |

**Table S1**. Most relevant clinical, biological, and treatment data for all the included patients.

Abbreviations : ECOG PS : Eastern Cooperative Oncology Group Performance Status; CRP : C-Reactive Protein; Hb : Haemoglobin; WBC : White Blood Count; PLT : Platelets; eGFR : estimated Glomerular Filtration Rate; IgG : Immunoglobulin G; VEGF : Vascular Endothelial Growth Factor; IL-6 : Interleukin-6; HIV : Human Immunodefiency Virus; CD : Castleman Disease; UCD : Unicentric Castleman Disease; iMCD-NOS : idiopathic Multicentric Castleman Disease; HHV-8 : Herpes Human Virus-8; HV : hyaline vascular; PC : plasmacytic; NA : Not Assessed; CR : Complete Response; SD : Stable Disease; PR : Partial Response; PD : Progression of Disease; KS : Kaposi Sarcoma; AHIA : Autoimmune haemolytic anaemia; ITP : immune thrombocytopenia; ES : Evans Syndrome; RT : Radiotherapy; CVP : Cyclophosphamide, Vincristine, Prednisone

* Sepsis of cutaneous origin (lower limb gangrene)

° Diagnosis was made 2 years after CD. § Diagnosis was made 1 year after CD. # Diagnosis was made 8 years after CD
