# Supplementary material for: Clinical Presentation, Treatment, and Outcomes of 28 Patients With Castleman Disease: A Retrospective Analysis of an Italian Cohort
Source: EJHaem. 2025 Nov 6;6(6):e70158. doi: 10.1002/jha2.70158 (PMC12591177; doi:10.1002/jha2.70158)
Supplement: Supplementary file 2 — Table S2. Histopathological grading for all iMCD‐NOS patient according to Fajgenbaum et al. Blood 129, 1646–1657 (2017). [file JHA2-6-e70158-s003.docx]

| **Case number** | **Diagnosis** | **Histology** | **Regressed GCs** | **FDC prominence** | **Vascularity** | **Hyperplastic GCs** | **Plasmacytosis** |
| --- | --- | --- | --- | --- | --- | --- | --- |
| 3 | iMCD-NOS | HV | 2 | 2 | 2 | 1 | 0 |
| 9 | iMCD-NOS | PC | 1 | 1 | 2 | 2 | 3 |
| 16 | iMCD-NOS | PC | 1 | 1 | 2 | 2 | 3 |
| 17 | iMCD-NOS | PC | 2 | 2 | 1 | 1 | 3 |
| 18 | iMCD-NOS | Mixed | 2 | 2 | 1 | 2 | 2 |
| 20 | iMCD-NOS | Mixed | 3 | 2 | 1 | 1 | 2 |
| 25 | iMCD-NOS | Mixed | 2 | 2 | 2 | 0 | 2 |
| 26 | iMCD-NOS | PC | 1 | 2 | 2 | 1 | 3 |
| 27 | iMCD-NOS | Mixed | 3 | 1 | 3 | 0 | 1 |
| 28 | iMCD-NOS | Mixed | 3 | 0 | 1 | 0 | 1 |

**Table S2**. Histopathological grading for all iMCD-NOS patient according to Fajgenbaum et al. *Blood* 129, 1646–1657 (2017).

Abbreviations: i-MCD-NOS : idiopathic Multicentric Castleman Disease; HV : hyaline vascular; PC : plasmacytic; GC : Germinal Center; FDC : Follicular dendritic cells; NA : Not Assessed-
