# Supplementary material for: Clinical Presentation, Treatment, and Outcomes of 28 Patients With Castleman Disease: A Retrospective Analysis of an Italian Cohort
Source: EJHaem. 2025 Nov 6;6(6):e70158. doi: 10.1002/jha2.70158 (PMC12591177; doi:10.1002/jha2.70158)
Supplement: Supplementary file 3 — Table S3. Summary of bone marrow characteristics, kappa/lambda nodal immunostaining, and BCR rearrangement analysis for patients with iMCD‐NOS and M protein, used for ruling out diagnoses other than Castleman disease or Castleman‐like lesions. [file JHA2-6-e70158-s001.docx]

| **Case N.** | **Age** | **Diagnosis** | **Histology** | **Monoclonal protein** | **Bone marrow biopsy at CD diagnosis** | **K/lambda restriction on nodal biopsy** | **CD30** | **BCR rearrangement** |
| --- | --- | --- | --- | --- | --- | --- | --- | --- |
| 17 | 50 | iMCD-NOS | PC | **IgG-lambda** | Normal | Lambda > k  K/lambda 1/20 | Scattered cells | Polyclonal |
| 18 | 60 | iMCD-NOS | mixed | **IgG-lambda + IgG-kappa** | Plasmacytic polyclonal infiltration (5%) | Lambda > k  polyclonal | Scattered cells | Monoclonal |
| 20 | 65 | iMCD-NOS | mixed | **IgG-lambda** | Normal | k >lambda  polyclonal | Scattered cells | Polyclonal |
| 26 | 40 | iMCD-NOS | PC | **IgA-lambda** | Normal | NA | Scattered cells | Polyclonal |
| 27 | 56 | iMCD-NOS | mixed | **IgG-kappa** | Plasmacytic polyclonal infiltration (10%) | k > lambda  polyclonal | Scattered cells | Polyclonal |

**Table S3.** Summary of bone marrow characteristics, kappa/lambda nodal immunostaining, and BCR rearrangement analysis for patients with iMCD-NOS and M protein, used for ruling out diagnoses other than Castleman disease or Castleman-like lesions.

IGVH gene rearrangements were investigated through PCR for FR1, FR2, and FR3 using IdentiClone®IGH Gene Clonality Assay (Invivoscribe, San Diego, California, USA) on FFPE samples. PCR products were subjected to fragment analysis and investigated using GeneScan Analysis Software (Thermo Fisher Scientific, Waltham, Massachusetts, USA). In all cases, IgG4+/IGG+ plasma cells ratio was <40%, except for case n. 17, which showed an increase in IGG4+ plasma cells.

Abbreviations: iMCD-NOS: idiopathic Multicentric Castleman Disease Not Otherwise Specified; plasmacytic variant; NA: not assessed; BCR : B-cell Receptor; PC plasmacellular
